# Argonine and Argentamine

**Published:** 1895-10-05

**Authors:** 


### THERAPEUTIC NOTES.

### ARGONINE AND ARGENTAMINE.

Caseinate of silver, or argonine, has been introduced as a substitute for nitrate of silver as a local application. A. Liebrecht[1], starting from the fact that solutions of albumen containing free alkali do not give a precipitate with the soluble salts of silver, concluded that it might be possible to obtain a soluble albuminous compound of silver with an alkali, but in which the alkali was not in a free state, and thus a compound which while being deprived of all caustic properties, would nevertheless preserve its bactericidal powers.

It is probable that the irritating effect of nitrate of silver on mucous membranes is deleterious, and that the beneficial effect of this salt depends on its bactericidal properties. Moreover, the insoluble compound formed by nitrate of silver with albumen militates against its efficiency as an antiseptic.

Argentamine is a solution of phosphate of silver in ethylene diamine. It is strongly alkaline, and acts as though it contained free alkali; it precipates albumen to some extent, and is strongly irritating.

A combination of silver with albumen, which is not alkaline and still possesses bactericidal properties, may be obtained by mixing a solution of a sodium compound of casein with silver nitrate, and precipitating with alcohol. The precipitate, after desiccation, is a fine white powder of neutral reaction, and is termed argonine.

Argonine is readily dissolved in warm water, and with some difficulty in cold. It is necessary to observe certain precautions in order to dissolve the substance in water. One should begin by vigorously shaking some argonine with a little cold water, so as to separate completely all the particles of the salt; then the vessel containing the mixture is heated over a water-bath heated to 90 deg., shaking it to hasten the solution. The watery solution, readily obtained in this manner in five minutes, is a slightly coloured opalescent liquid, and may be made in any strength up to 10 per cent.

Like all salts of silver, argonine, especially in solutions, is affected by light and should be kept in dark-coloured glass botties. Argonine is readily dissolved in albumen. Thus on shaking it up with serum, one can obtain a 10 per cent. solution in serum. Solutions containing albumen, as for example, white of egg, serum of bullock's blood, hydrocele fluid, are not precipitated by argonine, but if left for some time a scarcely perceptible deposit occurs. One cannot separate the silver by the ordinary reactions for salts of silver, thus chloride of sodium and alkalies do not give a precipitate in argonine solutions.

The therapeutic effects of argonine have been investigated by R. Mayer[2], who concludes that argonine exerts no caustic action on the mucous surface; in this respect it differs favourably from argentamine and nitrate of silver. With a view to test their power of penetration into tissues, Mayer placed these three salts of silver on solidified gelatine, which he placed in the dark, with the result that argonine displayed this property in the greatest degree.

Secondly, this author investigated the bactericidal properties of these three salts of silver with the following results:—

A.—Bacteria held in suspension in water (staphylococcus bact. coli, bacillus pyocyaneus, prodigiosus, tetragenus, cholera bacillus):—

1. Argentamine, in a 1:4,000 solution, is more efficient in its disinfectant power than argonine and nitrate of silver in the same strength.
2. With increasing concentration the antiseptic power of nitrate of silver increases more rapidly than that of argonine.
3. Argonine, in strengths of 1:750 or 1:1,000, is better tolerated in blennorrhagia than is argentamine in 1:4,000 solution, or than nitrate of silver 1:3,000. At the same time this solution of argonine is superior in its antiseptic power to the other two preparations of silver.
4. The addition of ammonia to argonine (0·3 gr.—0·6:100) increases the antiseptie power of argonine considerably.

B.—The same micro-organisms cultivated in albuminoid solutions.

In this case the disinfecting power of argonine (1:750) is equal to that of nitrate of silver (1:1,000) and argentamine (1:4,000).

Argonine in solution 1:750 destroys gonococci quite as rapidlly as does nitrate of silver 1:6,000, but ammoniacal argonine was the most potent disinfectant in every case. At the same time, the addition of ammonia renders it more irritating to mucous surfaces.

It would appear that the bactericidal action of silver depends[3] not on the insoluble compound of albumen and silver, but is produced by the action of the metal itself. It is found, too, that a solution of argonine injected subcutaneously produces death with the ordinary symptoms of acute metallic poisoning. After a corresponding injection of nitrate of silver the animal lived a week.

According to Aschner,[4] argentamine is well borne by the deeper portions of the urethra in solution 1 : 1,000, 1 : 500, and also in solution 1 : 250. The anterior portion of the urethra is less tolerant, and one should not inject solution of greater strength than 1 : 2,000 or 1 : 1,000.

Suppuration is increased, especially by strong solutions and only diminishes slowly after three or four days, and in every case astringents should be simultaneously used to prevent swelling of the mucosa. With these solutions used by means of Ultzmann's injector gonococci disappear by the tenth or twelfth day, *i.e.*, in a shorter time than is obtained by means of nitrate of silver. Argentamine, according to this author, penetrates the tissues, and is better in this respect than nitrate of silver.

[1] Ther. Mutsh., No. VI., 1895. [2] Zeit. f. Hyg. ur Infectkuht, Bd. xx. p. 1., 1895. [3] Lèsbrecht cited Med. Chirur., 1895, No. 7, p. 280. [4] Orv. hetil., 1895, No. 11-13. Ther. Wehushft, 1895, No. 22., p. 464-465.

